# Clinicopathologic factors associated with recurrence in parotid carcinoma^☆^

**DOI:** 10.1016/j.bjorl.2017.08.003

**Published:** 2017-08-25

**Authors:** Dong Hoon Lee, Tae Mi Yoon, Joon Kyoo Lee, Sang Chul Lim

**Affiliations:** a Chonnam National University, Medical School, Department of Otolaryngology-Head and Neck Surgery, Hwasun, South Korea; b Chonnam National University, Hwasun Hospital, Hwasun, South Korea

**Keywords:** Parotid gland, Parotid cancer, Recurrence, Surgical procedures, Radiotherapy, Glândula parótida, Câncer de parótida, Recorrência, Procedimentos cirúrgicos, Radioterapia

## Abstract

**Introduction:**

Parotid carcinomas have varying histological types and diverse biologic behaviors. Establishing an adequate treatment plan and predicting recurrence is important.

**Objective:**

To analyze the risk factors associated with recurrence in our 5 year experience with 30 cases of primary parotid carcinoma undergoing surgery at a single institute.

**Methods:**

From January 2009 to December 2013, 30 patients with surgical treatment of parotid carcinoma were identified based on their medical records.

**Results:**

The 30 patients were comprised of 17 males and 13 females. Among 11 patients with T4 tumors, seven patients had recurrence. Among seven patients with cervical nodal metastasis, all patient except one had recurrence. Clinically late stages (stage III and IV) showed more common recurrence than early stage (stage I and II) lesions. Lymphovascular invasion was seen in 5 patients, and all patients had recurrence. Among 11 patients with extracapsular spread, 7 patients had recurrence. In 17 patients with high grade carcinomas, ten patients had recurrence. In 13 patients with low grade carcinomas, no patients experienced recurrence.

**Conclusion:**

T- and N-stage, clinical stage, lymphovascular invasion, extracapsular spread, and histopathologic grade correlate significantly with recurrence in parotid carcinoma.

## Resumo

**Introdução:**

Os carcinomas da parótida têm diferentes tipos histológicos e comportamentos biológicos diversos. O estabelecimento de um plano de tratamento adequado e a previsão de recorrência são muito importantes.

**Objetivo:**

Analisar os fatores de risco associados à recorrência em nossa experiência de cinco anos com 30 casos de carcinoma parotídeo primário submetidos a cirurgia em uma única instituição.

**Método:**

De janeiro de 2009 a dezembro de 2013, 30 pacientes com tratamento cirúrgico de carcinoma parotídeo foram identificados com base nos prontuários.

**Resultados:**

Entre os 30 pacientes, 17 eram homens e 13, mulheres. Dos 11 pacientes com tumores T4, sete apresentaram recorrência. Entre sete pacientes com metástase em linfonodo cervical, todos, exceto um, apresentaram recorrência. Lesões em estágios clínicos tardios (III e IV) apresentaram recorrência mais comumente do que as dos estágios iniciais (I e II). A invasão linfovascular foi observada em cinco pacientes e todos os cinco apresentaram recorrência. Entre 11 pacientes com disseminação extracapsular, sete apresentaram recorrência. Dos 17 pacientes com carcinomas de alto grau, dez apresentaram recorrência. Em 13 pacientes com carcinomas de baixo grau, nenhum apresentou recorrência.

**Conclusão:**

Estágios T e N, estágio clínico, invasão linfovascular, disseminação extracapsular e grau histopatológico correlacionam-se de maneira significante com recorrência do carcinoma de parótida.

## Introduction

Parotid carcinoma is an uncommon malignancy and constitutes 1%–3% of all head and neck cancers.^1^, ^2^, ^3^, ^4^, ^5^ Parotid carcinomas have varying histological types and diverse biologic behaviors.^1^, ^2^, ^3^, ^4^, ^5^ Treatment of parotid carcinoma remains challenging because of its relative rarity, unpredictable biological behavior, and risk of recurrence.^1^, ^2^, ^5^, ^6^

Therefore, establishing an adequate treatment plan and predicting recurrence is important.^5^ In general, surgery is the treatment of choice for all parotid tumors and postoperative radiotherapy (RT) is applied as supplementary treatment.^2^, ^4^, ^5^, ^6^ However, there have been few published reports about predicting recurrence.^5^ The identification of factors associated with recurrence is of paramount relevance for treatment of parotid carcinomas.

The aim of this study was to analyze risk factors associated with recurrence in our 5 year experience with 30 cases of primary parotid carcinoma undergoing surgery at a single institute.

## Methods

After obtaining approval from the Institutional Review Board of our Hospital (CNUHH-2016-134), a retrospective review was performed to evaluate patients with surgical treatment of parotid carcinoma at the hospital's Department of Otolaryngology-Head and Neck Surgery from January 2009 to December 2013. Thirty patients with surgical treatment of parotid carcinoma were identified based on their medical records. Clinicopathologic data of parotid carcinomas were reviewed including age, sex, symptoms, duration of symptoms, lymph node status, fine-needle aspiration cytology (FNAC), overall stage, histopathologic results, surgical procedures and complications. The 2010 version of the TNM staging system of the American Joint Committee on Cancer was used for clinical staging.^7^

All patients had computed tomography (CT) scanning performed before surgery to assess the extent of the lesions and to aid in treatment planning. Positron emission tomography-CT (PET-CT) was performed for those patients with malignancy by FNAC. All patients except two underwent FNAC.

The type and extent of surgery performed depended on the pre-operative diagnosis, primary site, and surgeon's clinical judgment. All patients underwent macroscopically complete oncologic resection. Superficial parotidectomy was performed if a small carcinoma was located in the superficial lobe. Total parotidectomy was performed if the carcinoma was in the deep lobe or in a tumor diagnosed as malignant by FNAC. Radical parotidectomy, involving removal of all parotid tissue as well as sacrifice of the facial nerve, was performed if the facial nerve was invaded by carcinoma or if preoperative facial nerve function was impaired in the presence of malignant disease. Neck dissection was performed if enlarged neck lymph nodes were found by preoperative evaluation, such as FNAC and radiologic examination.

Postoperative management, such as RT and concurrent chemoradiotherapy (CCRT), were dependent on tumor stage and histological grade. Postoperative radiation therapy had been performed for patients with lymph node metastasis, high grade carcinoma, positive surgical margin, and high clinical stage. Drainage was performed and maintained by aspiration. All cases of parotid carcinoma were confirmed histopathologically. The complication of postoperative facial palsy was evaluated by House Brackman grade. Intraoperative facial nerve monitoring was typically used. The overall survival period was determined from the date of surgery to the date of death or the date of the last visit.

SPSS version 20.0 software was used to conduct statistical analyses. Fisher's exact test was used to analyze the association between recurrence/surgical margin and clinicopathological parameters. Survival rates were calculated using the Kaplan–Meier method with the log-rank test. Multivariate analysis to survival was conducted using Cox proportional hazards regression model. Statistical significance was defined as a *p*-value <0.05.

## Results

This group of 30 patients included 17 (56.7%) males and 13 (43.3%) females. The age of the patients ranged between 23 and 83 years with a mean of 62.6 ± 14.1 years. All patients except 2 (28/30, 93.3%) presented with a slowly enlarging mass within the parotid gland. The remaining two patients were incidentally diagnosed by PET-CT. Of the 30 lesions, 13 parotid carcinomas (43.3%) were located in the right parotid gland and 17 parotid carcinomas (56.7%) in the left parotid gland. The majority of the lesions were asymptomatic. The duration of symptoms ranged from 1–120 months with a mean of 12.2 ± 22.7 months.

Thirteen patients (43.3%) were classified as having T1 tumors, 5 patients (16.7%) had T2 tumors, 1 patient (3.3%) had T3 tumors, and 11 patients (36.7%) had T4 tumors. Seven cases (23.3%) had lymph node metastasis, and no patients had distant metastasis. The clinical staging showed that 12 patients (40.0%) were classified as stage I, 4 patients (13.3%) as stage II, 1 patient (3.3%) as stage III, and 13 patients (43.3%) as stage IV.

The most common surgical procedure was superficial parotidectomy (*n* = 13, 43.3%). Followed by total parotidectomy (*n* = 12, 40.0%) and radical parotidectomy (*n* = 5, 16.7%). Neck dissection was performed in 15 patients (50%).

Eleven patients had salivary ductal carcinoma, followed by mucoepidermoid carcinoma (*n* = 7), squamous cell carcinoma (*n* = 3), carcinoma ex pleomorphic adenoma (*n* = 2), acinic cell carcinoma (*n* = 2), adenoid cystic carcinoma (*n* = 2), epithelial-myoepithelial carcinoma (*n* = 1), lymphoepithelial carcinoma (*n* = 1), and polymorphous low grade adenocarcinoma (*n* = 1). Fourteen patients underwent postoperative RT. Seven patients underwent postoperative CCRT.

Among 28 patients who underwent FNAC, 15 patients were diagnosed with parotid carcinoma, but the remaining 13 patients were failed in preoperative diagnosis. FNAC had a diagnostic sensitivity of 53.6%, diagnostic specificity of 0%, positive-predictive value of 100%, negative-predictive value of 0% and accuracy of 53.6% for diagnosing benign parotid tumors. No specific complications were observed after FNAC.

In histopathologic results, surgical margins were negative in 16 (53.3%), positive in 4 (13.3%), and close (<5 mm) in 10 patients (33.3%) (Table 1). Perineural invasion, lymphovascular invasion, and extracapsular spread were seen in 7, 5, and 11 patients, respectively. Histopathologic grading revealed that 13 patients (43.3%) were classified as having low grade carcinoma, no patients had intermediate grade carcinoma, and 17 patients (56.7%) had high grade carcinoma.

**Table 1:** Summary of clinicopathologic factors associated with surgical margin.

Factors	Surgical margin		*p*-value
	Negative (*n* = 16)	Close or positive (*n* = 14)	
*T stage*			0.257
T1, T2, T3	12	7	
T4	4	7	

*N stage*			0.675
N0	13	10	
N1, N2	3	4	

*Stage*			0.141
I, II	11	5	
III, IV	5	9	

*Lymphovascular invasion*			0.157
Negative	15	10	
Positive	1	4	

*Extracapsular spread*			0.007
Negative	14	5	
Positive	2	9	

*Histologic grade*			0.484
Low grade	8	5	
High grade	8	9	

*Recurrence*			0.122
No	13	7	
Yes	3	7	

Nine (30.0%) patients had facial nerve palsy. Four of these patients presented with spontaneous improvement within 3 months of surgery. The remaining 5 patients had complete facial nerve palsy (House Brackman Grade VI), because in all patients the facial nerve was deliberately sacrificed due to its involvement with the malignant tumor.

Ten patients (33.3%) experienced recurrence (Table 2). Sites of recurrence were local in 6 patients and distant in 7 patients. Three patients had both local and distant metastases. The sites of distant metastasis were lung and liver. Among surviving patients, one patient was alive with recurrent carcinoma in the absence of any other treatment at the last follow-up.

**Table 2:** Summary of clinicopathologic factors associated with recurrence.

Factors	Recurrence		*p*-value
	Yes (*n* = 10)	No (*n* = 20)	
*T stage*			0.042
T1, T2, T3	3	16	
T4	7	4	

*N stage*			0.001
N0	4	19	
N1, N2	6	1	

*Stage*			0.004
I, II	1	15	
III, IV	9	5	

*Lymphovascular invasion*			0.019
Negative	5	20	
Positive	5	0	

*Extracapsular spread*			0.042
Negative	3	16	
Positive	7	4	

*Chemoradiation*			0.013
Yes	10	11	
No	0	9	

*Histologic grade*			0.003
Low grade	0	13	
High grade	10	7	

**Figure 1:**
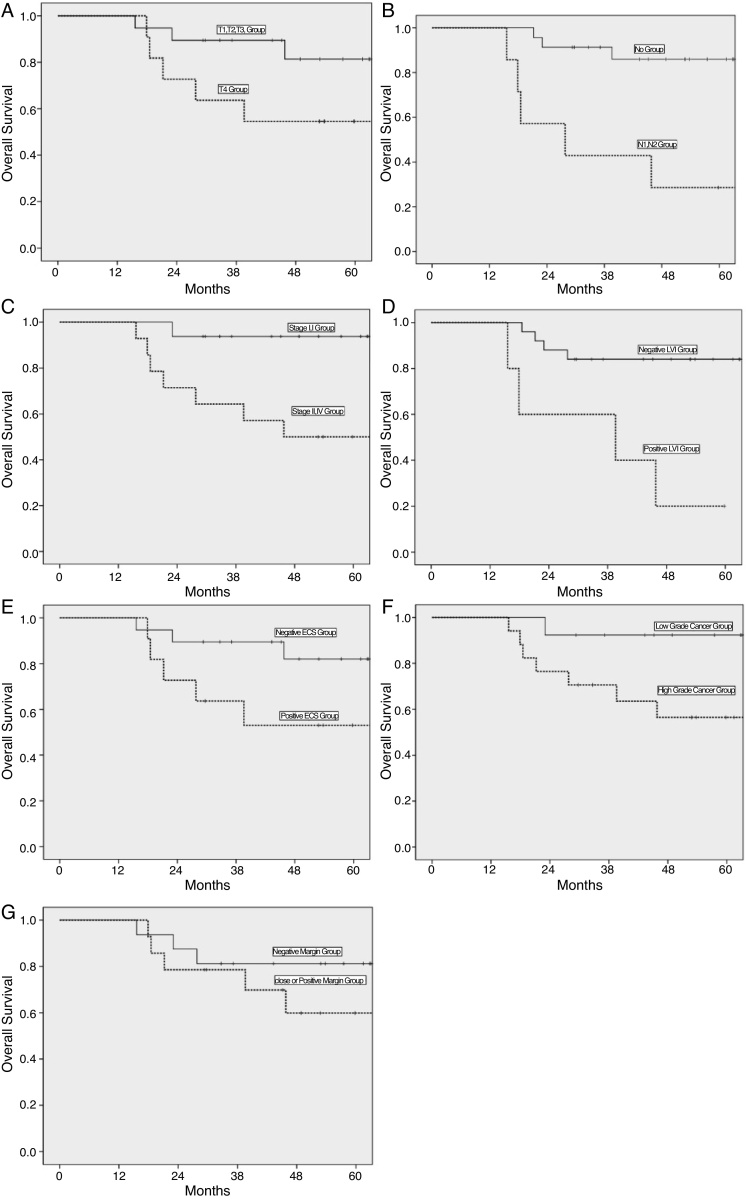
Comparison of survival according to clinicopathologic factors. (A) T-stage (*p* = 0.097); (B) N-stage (*p* = 0.001); (C) clinical stage (*p* = 0.011), (D) lymphovascular invasion (*p* = 0.002); (E) extracapsular spread (*p* = 0.078); (F) histopathologic grade (*p* = 0.049); (G) surgical margin (*p* = 0.313).

The mean follow period after surgery was 56.7 ± 16.0 months with a range of 29–86 months. The 2, 3 and 5 year overall survival rate were 80%, 71%, and 71%, respectively. According to Kaplan–Meier method, N-stage, clinical stage, lymphovascular invasion, histopathologic grade correlated significantly with survival in parotid carcinoma (Fig. 1). In the Cox multivariate regression analysis, only N-stage was associated with survival in this study (Table 3, Table 4). Therefore, N-stage was the most significant factor in patients with parotid carcinoma.

**Table 3:** Cox multivariate regression of the association with survival.

Covariate	*p*-value	Hazard ratio	95% Confidence interval	
			Lower	Upper
Age	0.784	0.988	0.903	1.080
T stage	0.626	0.495	0.029	8.362
N stage	0.045	9.462	1.052	85.093
Extracapsular spread	0.367	4.334	0.179	105.162
Surgical margin	0.973	0.971	0.175	5.377

**Table 4:** Logistic regression of the association with survival.

Covariate	*p*-value	Exp(*B*)	95% Confidence interval	
			Lower	Upper
Age	0.576	1.042	0.902	1.203
T stage	0.675	1.949	0.087	43.888
N stage	0.050	24.597	0.993	609.112
Extracapsular spread	0.470	4.722	0.070	318.258

## Discussion

Previous studies have suggested several clinical and histological factors associated with recurrence of parotid carcinoma, such as TNM staging system, clinical stage, perineural or lymphovascular invasion, extracapsular spread, positive surgical margin, and histological grade.^1^, ^4^, ^5^, ^8^, ^9^, ^10^, ^11^, ^12^ In this study, T- and N-stage, clinical stage, lymphovascular invasion, extracapsular spread, histopathologic grade were found to be factors associated with recurrence (Table 2).

Among 11 patients with T4 tumors, seven patients experienced recurrence. Among 19 patients with T1, T2, and T3 tumors, only 3 patients had recurrence. T4 tumors experienced more recurrence than other T stage tumors (*p* < 0.05). Among 7 patients with cervical nodal metastasis (N1, N2), all patients except one had recurrence. There was a significant difference between cervical nodal metastasis and recurrence of parotid carcinoma (*p* < 0.05). In clinical staging, late stages (stage III and IV) showed more recurrence more frequently than early stages (stage I and II) lesions (*p* < 0.05). In this study, T4 tumors, cervical lymph node metastasis, and high clinical stage were prognostic factors related to the recurrence of parotid carcinoma.

In this study, extracapsular spread was found to be factors associated with surgical margin (Table 1). In the histopathologic results, lymphovascular invasion were seen in 5 patients, and all patients had recurrence. Among 11 patients with extracapsular spread, seven patients had recurrence. Among 19 patients without extracapsular spread, only 3 patients had recurrence. In 17 patients with high grade carcinomas, 10 patients had recurrence. In 13 patients with low grade carcinomas, no patients experienced recurrence. In this study, the presence of lymphovascular invasion and extracapsular spread, as well as high grade carcinomas were prognostic factors related to the recurrence of parotid carcinoma. In particular, all 5 patients with lymphovascular invasion had distant metastasis. Among 5 patients, 2 patients had both local and distant metastases. In addition, there was no recurrence of low grade parotid carcinoma in this study(Table 3, Table 4).

Parotid carcinoma usually requires a combination of treatment modalities.^1^, ^6^, ^7^, ^8^, ^9^, ^10^, ^11^, ^12^, ^13^ Surgical resection followed by RT or CCRT improves loco-regional control and overall survival.^3^, ^6^, ^13^ Our indications of postoperative RT include positive or close margins, high grade carcinomas, perineural or lymphovascular invasion, and cervical lymph node metastasis. Of our patients with low grade carcinomas (*n* = 13), 4 patients had been well without recurrent combined modality treatment with surgery followed by postoperative RT because of close surgical margins. In high grade carcinomas (*n* = 17), ten patients had surgery and postoperative RT, and 7 patients underwent surgery and postoperative CCRT. Simultaneous neck dissection is recommended when neck metastasis is clinically detected or when a histologically high grade malignancy, high stage, facial palsy or extraparotid invasion are diagnosed.^4^ In our sample, radical neck dissection was performed in seven cases of parotid carcinomas with cervical lymph node metastasis, and elective neck dissection was performed in eight N0 parotid carcinoma patients.

In this study, significant factors associated with recurrence were T- and N-stage, clinical stage, lymphovascular invasion, extracapsular spread, and histopathologic grade. Whereas, extracapsular spread was found to be factors associated with surgical margin. However, the limiting factors of this study are the small sample size and retrospective review. Further studies involving molecular markers are necessary to provide a better understanding of the biological mechanisms of parotid carcinoma recurrence.

## Conclusion

T- and N-stage, clinical stage, lymphovascular invasion, extracapsular spread, and histopathologic grade correlate significantly with recurrence in parotid carcinoma. Among them, N-stage was the most significant factor in this study. High risk patients require aggressive initial surgery with postoperative RT and regular long-term follow-up.

## Conflicts of interest

The authors declare no conflicts of interest.
